# Targeting the activin/myostatin - actrii pathway to preserve skeletal muscle mass in obesity: mechanistic insights and therapeutic perspectives

**DOI:** 10.1007/s11154-026-10033-w

**Published:** 2026-04-09

**Authors:** Giuseppe Lisco, Anna De Tullio, Olga Eugenia Disoteo, Palma Dicorato, Anna Tortora, Vincenzo De Geronimo, Vincenzo Triggiani

**Affiliations:** 1 Interdisciplinary Department of Medicine, School of Medicine, University of Bari Aldo Moro, Piazza Giulio Cesare 11, Bari, 70124 Italy; 2 Unit of Endocrinology, Diabetology, Dietetics and Clinical Nutrition, Sant Anna Hospital, San Fermo della Battaglia, 22020 Italy; 3 Unit of Endocrinology and Metabolic Diseases, Local Health Authority of Taranto, Socio- Health District 5, Martina Franca, 74015 Italy; 4 Unit of Endocrinology and Diabetology, Ospedale Riuniti San Giovanni di Dio e Ruggi D’Aragona, Salerno, 81131 Italy; 5 Unit of Endocrinology, Clinical Diagnostic Center Morgagni, Catania, 95100 Italy

**Keywords:** Obesity, Sarcopenia, Glp-1 receptor agonists, Tirzepatide, Bimagrumab

## Abstract

Skeletal muscle deterioration is a relevant complication of obesity associated with poor physical function, impairment of metabolic homeostasis, and worse lifelong outcomes. The phenomenon can be accentuated by weight loss, especially in patients with poor skeletal muscle health at baseline (e.g., sarcopenic obesity) and those experiencing rapid and significant slimming. Preserving and even strengthening skeletal muscle mass during weight loss would be a novel therapeutic target, as disproportionate fat to skeletal muscle mass loss reduces metabolic rate, physical performance, and long-term health benefits. Anti-obesity pharmacotherapies, especially glucagon-like peptide 1 receptor agonists and tirzepatide, preferentially reduce fat mass while partially sparing skeletal muscle, though significant lean mass loss has been described in individuals with pronounced weight reduction. The review aims to elucidate innovatively the mechanistic role of the activin/myostatin receptor pathways in obesity-associated skeletal muscle atrophy and explore therapeutic strategies targeting this axis to preserve muscle mass, quality, and function. Targeted inhibition of the activin/myostatin pathway is a novel strategy to enhance muscle trophism. Activin receptor (IIA/IIB) blockade prevents the small mothers against decapentaplegic homolog 2/3-mediated muscle catabolism, promoting myofibrillar synthesis, myoblast differentiation, and hypertrophy. Bimagrumab - a human anti-activin receptor antibody - consistently increases skeletal muscle mass and improves body composition across populations, including sarcopenic adults, patients with disuse atrophy, and individuals with obesity. While gains in strength are variable, this approach represents a promising adjunct to lifestyle and pharmacologic interventions. Integrating skeletal muscle preservation into obesity management may optimize functional, metabolic, and clinical outcomes, representing a paradigm shift in comprehensive care.

## Introduction

Obesity is a widespread, multifactorial, chronic, and relapsing disease characterized by excessive, abnormal and/or dysfunctional adiposity accumulation [1]. The Lancet obesity Commission has recently provided an updated consensus document addressing key issues related to obesity from an integrated clinical, scientific, ethic, and socio-political perspective [2]. The Commission conceptualizes obesity as a disease continuum, distinguishing between clinical obesity - defined by the presence of established obesity-related complications - and preclinical obesity, characterized by excess adiposity in the absence of overt chronic complications [3]. Adiposity contributes to mechanical and functional impairments, adversely affecting the osteoarticular, musculoskeletal, and respiratory systems. In parallel, adipose tissue dysfunction - through chronic low-grade inflammation and hormonal and adipokine dysregulation - drives the development of cardiovascular, renal, gastrointestinal, and reproductive complications, as well as endocrine and metabolic disorders. Moreover, psychosocial stigma experienced by individuals living with obesity represents an additional burden and it is strongly associated with depression, anxiety, and body image dissatisfaction [4]. Last, obesity is associated with an increased risk of several site-specific, obesity-related cancers [5].

Ongoing research is aimed to identify the most accurate methods for the assessment of adiposity excess. For decades, body mass index (BMI) has been widely used as a surrogate of body weight excess and adiposity [6], and as a pragmatic tool to associate adipose tissue expansion with a broad range of obesity-related outcomes [7]. However, BMI does not capture body fat distribution or qualitative aspects of adipose tissue. For this reason, additional anthropometric measures should be utilized to improve the estimation of adiposity burden and its regional distribution [8]. In routine clinical practice, the combined use of BMI and at least one complementary anthropometric parameter - such as waist circumference or waist-to-hip ratio - is therefore recommended to provide a more refined assessment of total adiposity and its distribution. More recently, direct methods for body composition assessment have been increasingly adopted in individuals with obesity. These techniques offer greater accuracy in quantifying adiposity enlargement and show stronger associations than indirect indices (e.g., BMI) with obesity-related complications and disease burden [9, 10]. Furthermore, body composition analysis yields comprehensive information on individual body compartments, including fat-free mass (FFM), skeletal muscle mass (SMM), and total body water, which cannot be reliably estimated using conventional anthropometric measures alone [11].

Interest in skeletal muscle health among individuals with obesity has increased substantially in recent years. Compared with healthy controls, patients with obesity exhibit significantly lower SMM, and approximately 20% of adults living with obesity in Western countries have concomitant sarcopenia [12]. Sarcopenia is a complex, multifactorial clinical condition in which genetic and metabolic determinants, chronic low-grade inflammation, malnutrition, unhealthy lifestyle behaviors, and aging drive a progressive decline in SMM and muscle strength [13]. This process ultimately results in impaired physical performance, functional limitations, increased risk of falls, and elevated mortality. Despite substantial heterogeneity in the diagnostic criteria and assessment methods used to define sarcopenic obesity (SO) [14], the overall estimated prevalence is approximately 14%, with markedly higher rates observed in specific subgroups, including sedentary individuals (17%), those with two or more chronic comorbidities (19%), individuals with functional impairment (33%), and those with cognitive impairment (35%) [15]. In SO, there is a coexistence of sarcopenia - diagnosed when appendicular skeletal muscle index is < 7 kg/m² in men and < 5.5 kg/m² in women (assessed with dual-energy X-ray absorptiometry) with evidence of low handgrip strength (< 27 kg men and < 16 kg women) or gait speed < 0.8 m/s - and excess adiposity, as usually defined by BMI ≥ 30 kg/m² or altered body composition (body fat mass > 30% men and > 40% women) [16]. Notably, SO is frequently identified among candidates for bariatric surgery, with reported prevalence rates of up to 23%, underscoring the role of severe body weight enlargement and adipose tissue dysfunction as major contributors to skeletal muscle deterioration [17]. In this context, the European Society for Clinical Nutrition and Metabolism and the European Association for the Study of Obesity have advocated for structured diagnostic pathways to identify SO. Specifically, assessment of skeletal muscle strength using functional tests is recommended as the initial step to detect muscle function impairment. This should be followed by comprehensive body composition analysis to confirm the diagnosis, based on the coexistence of reduced SMM and strength in the presence of excess adiposity [16]. Beyond baseline conditions, individuals with obesity commonly experience a clinically relevant loss of FFM and skeletal muscle during weight reduction [18]. This phenomenon is particularly pronounced in patients undergoing substantial weight loss induced by bariatric surgery [19] or by highly effective anti-obesity pharmacotherapies [20], with potentially deleterious consequences for muscle strength and overall physical performance.

While the current body of evidence has clearly delineated the clinical burden of muscle loss in obesity and during caloric restriction - supporting strategies such as high-protein diets and resistance exercise to attenuate skeletal muscle mass depletion - it has largely neglected the underlying molecular mechanisms that drive this process. In particular, more attention should be paid to the activin/myostatin signaling axis, a critical regulator of muscle homeostasis that appears to play a central role in the pathogenesis of SO and in its potential exacerbation during concomitant anti-obesity pharmacotherapy [21]. The activin/myostatin pathway is upregulated in individuals with excess adiposity. Beyond inhibiting skeletal muscle hypertrophy, its activation promotes adipose tissue expansion, fibrotic remodeling, and insulin resistance. These pleiotropic effects may converge to impair skeletal muscle quality and induce maladaptive structural remodeling of the muscle-tendon unit, thereby amplifying functional decline in such a high-risk population [22]. Apart from highlighting the main pathophysiological mechanisms underlying skeletal muscle deterioration associated with body weight excess, the appropriate monitoring and interpretation of body composition components, and the importance of preserving skeletal muscle health in patients with obesity, particularly during weight loss, the review fills this gap by synthesizing emerging evidence on activin receptor type IIA and IIB (ActRII)-targeted therapies, offering a translational framework for muscle-preserving obesity management that integrates molecular insights with clinical trial outcomes to guide precision interventions.

## Pathophysiology of skeletal muscle wasting in obesity

The age-related decline in SMM, strength, and function is a progressive and involuntary physiological process. From approximately 30 years of age, SMM and strength decrease by an estimated 3–8% per decade [23], with an accelerated rate of decline after the age of 65 [24]. The loss of motoneurons and the consequent denervation of muscle fibers - leading to reductions in both fiber number and cross-sectional area - represent key biological mechanisms underlying age-related skeletal muscle wasting [25]. In addition to neurogenic factors, aging profoundly impairs skeletal muscle regenerative capacity. This impairment is particularly evident in type II muscle fibers, in which the overall number, proliferative potential, and differentiation capacity of satellite cells are significantly reduced [26].

Sustained alterations in skeletal muscle oxidative metabolism are also observed in obesity [27]. Mitochondrial dysfunction arises from impaired autophagy, increased susceptibility to oxidative stress - partly attributable to reduced age-related synthesis of peroxiredoxin-3 - and partial failure of the mitochondrial respiratory chain [28]. Mitochondrial dysfunction promotes the excessive generation of reactive oxygen species, which in turn induce mitochondrial DNA damage and lipid bilayer disruption, thereby further exacerbating mitochondrial impairment. Oxidative stress associated with mitochondrial dysfunction leads to overexpression of pyruvate dehydrogenase kinase 4 in skeletal muscle cells, which contributes to insulin resistance and promotes a metabolic shift toward fatty acid rather than glucose utilization, global energetic inefficiency, and amplification of oxidative stress [29]. Beyond alterations in mitochondrial bioenergetics, marked impairments in mitochondrial biogenesis and mitophagy have also been documented in sarcopenic skeletal myocytes, resulting in defective mitochondrial turnover and compromised organelle renewal [30].

Chronic systemic inflammation - characterized by elevated circulating levels of interleukin-6 (IL-6), interleukin-1β (IL-1β), and tumor necrosis factor-α (TNF-α) - is a hallmark of dysfunctional obesity, with higher cytokine concentrations reflecting a greater adiposity-related disease burden [31]. SMM and muscle strength are inversely correlated with circulating levels of pro-inflammatory cytokines. Within skeletal muscle cells, chronic inflammation - together with lipotoxicity, insulin resistance, and oxidative stress - suppresses activation of the mammalian target of rapamycin (mTOR) signaling pathway, resulting in impaired myofibrillar protein synthesis and accelerated muscle protein breakdown [32].

**Fig. 1:**
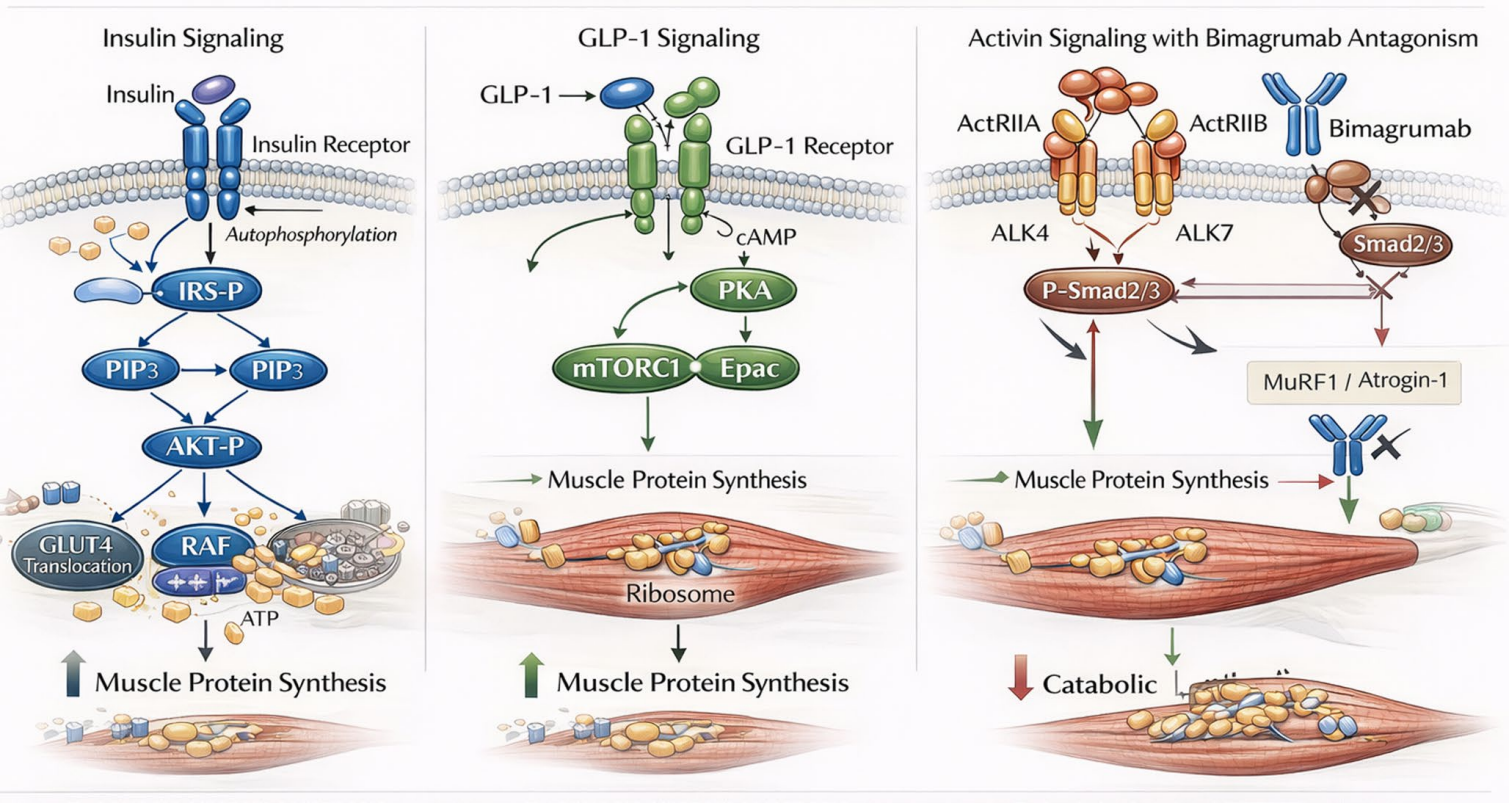
Illustration summarizing the signaling pathways of insulin, glucagon-like peptide 1, and activin/myostatin and their role in the regulation of myofibrillar synthesis and skeletal muscle trophism. Bimagrumab, an activin receptor antagonist, inhibits the activin signaling and MuRF1/Atrogin-1-dependent muscle protein breakdown, ultimately promoting considerable skeletal muscle hypertrophy [33–38]. Abbreviations: Phosphorylated Insulin Receptor Substrate, IRS-P; Phosphatidylinositol 3,4,5-trisphosphate, PIP3; Phosphorylated Protein Kinase B, AKT-P; GLUcose Transporter 4, GLUT4; Rapidly Accelerated Fibrosarcoma (pathway), RAF; Adenosine-Triphosphate, ATP; Glucagone-Like Peptide-1, GLP-1; Cyclic-Adenosine Monophosphate, cAMP; Protein Kinase A, PKA; mammalian Target of Rapamycin Complex1, mTORC1; Exchange Protein Activated by cAMP, Epac; Activin Receptor type II, ActRII; Small Mothers Against Decapentaplegic homolog, Smad; Anaplastic Lymphoma Kinase, ALK; Muscle RING-finger (protein), MuRF

Specific consideration should also be given to myokines. Myokines play a pivotal role in regulating skeletal muscle homeostasis, repair, and renewal [33], as well as in mediating crosstalk between myocytes and other organs, including adipose tissue [34]. Patients with obesity, insulin resistance, and type 2 diabetes (T2D) typically exhibit reduced circulating levels of several key myokines - such as irisin, decorin, myonectin, and fibroblast growth factor 19 - which are known to promote myofibrillar protein synthesis, suppress muscle protein catabolism, and restore mitochondrial turnover and metabolic efficiency, ultimately supporting skeletal muscle growth [35]. Follistatin is a liver-derived endogenous inhibitor that binds myostatin and modulates skeletal muscle growth by antagonizing myostatin activity [36]. Myostatin is an endogenous myokine that exerts a potent inhibitory signal on muscle growth and hypertrophy. By neutralizing myostatin, follistatin facilitates myofibrillar protein synthesis and skeletal muscle hypertrophy, as observed in the postprandial state or following protein intake, conditions in which circulating follistatin levels increase and closely correlate with serum insulin-like growth factor 1 (IGF-1) and insulin concentrations. Activin A and myostatin bind to ActRII, thereby promoting muscle catabolism through activation of the p38β mitogen-activated protein kinase-ubiquitin-proteasome pathway [37]. Insulin resistance and related chronic metabolic conditions - by altering circulating concentrations of these molecules and/or impairing peripheral responsiveness - attenuate both serum follistatin levels and its hypertrophic effects on skeletal muscle, thereby contributing to the development of sarcopenia (Fig. 1) [38].

Obesity-related hormonal imbalance contributes substantially to the development of sarcopenia. Beyond insulin resistance and the effects of pro-inflammatory cytokines, hyperleptinemia - known to promote skeletal muscle insulin resistance - and reduced adiponectin levels - which impair skeletal muscle utilization of energy substrates and ultimately compromise myofibrillar protein synthesis - individuals with obesity also exhibit lower circulating levels of growth hormone (GH) and total testosterone compared with healthy, age-matched controls [39]. This hormonal imbalance, which normally develops as part of the aging process, appears to occur earlier and with greater severity in patients with obesity than in normal-weight individuals. As a result, body composition is adversely affected, with a reduction in FFM and SMM, and a concomitant increase in fat mass (FM). These changes reflect a premature, profound, and persistent loss of the anabolic actions of GH and testosterone on both adipose tissue and skeletal muscle cells. GH promotes triglyceride breakdown, increases circulating free fatty acids, and modulates key enzymes and lipid droplet-associated proteins, and promotes adipokine secretion (adiponectin) [40]. GH and IGF-1 regulate preadipocyte proliferation, differentiation, and senescence while, in skeletal muscle, they act synergistically to enhance protein synthesis, limit proteolysis, and support muscle repair and regeneration [41]. Obesity, which is characterized by relative GH deficiency, chronic low-grade inflammation, and insulin resistance, dysregulated GH signaling may enhance catabolic responses during weight loss [42]. At the same time, testosterone is a key regulator of body composition, with low circulating levels being associated with reduced FFM and, particularly, SMM, and increased FM, particularly visceral adiposity. Testosterone replacement in hypogonadal men increases FFM and reduces total and visceral fat, reflecting true body recompositing rather than weight gain [43]. At the molecular level, testosterone promotes skeletal muscle anabolism via androgen receptor-mediated activation of the IGF-1/protein kinase B (AKT)/mTOR axis and inhibition of myostatin signaling. It also enhances erythropoiesis-mediated oxygen delivery to skeletal muscle, nitroxide, synthesis, mitochondrial biogenesis, ultimately enhancing anabolism and blunting catabolism [44]. In adipose tissue, testosterone suppresses adipogenesis and enhances lipolysis - thus contributing to a metabolically favorable phenotype - by inhibiting preadipocyte differentiation and promoting lipolysis through modulation of lipoprotein lipase activity and β-adrenergic signaling [45]. Testosterone also influences mesenchymal stem cell fate, favoring myogenic over adipogenic lineage commitment. Inadequate protein intake and physical inactivity are common lifestyle-related conditions in individuals with obesity and may contribute to sarcopenia both directly and indirectly, including through further disruption of hormonal homeostasis. Collectively, these mechanisms converge on downstream inhibition of the mTOR signaling pathway, in a manner similar to that observed with chronic exposure to pro-inflammatory cytokines [46].

## The importance of preserving skeletal muscle mass in obesity

Weight loss represents a key therapeutic goal in individuals with overweight or obesity [47], and preservation of FFM - particularly skeletal muscle - during FM reduction confers substantial clinical benefits [48]. Preferential loss of FM relative to FFM improves glucose metabolism, preserves resting metabolic rate [48], enhances physical performance and exercise tolerance, improves quality of life, and ultimately reduces all-cause mortality [49]. However, loss of FFM is largely unavoidable, as it represents an adaptive response to calorie-restrictive diets driven by metabolic and compensatory hormonal mechanisms [50]. Skeletal muscle accounts for approximately 10% or more of total weight loss [51]. This loss can be attenuated through adequate protein supplementation, with more pronounced benefits when combined with regular physical exercise compared with the absence of exercise [52, 53]. Pronounced weight loss is associated with an increased risk of substantial FFM loss (approximately − 8 kg) and SMM loss (around − 3 kg), as reported in several post-bariatric surgery trials [54, 55], and among individuals on anti-obesity medications [56]. This issue has important practical implications, as current recommendations emphasize the need to induce and maintain sufficient weight loss to prevent the onset or progression of obesity-related complications. Indeed, accumulating evidence indicates that greater weight loss is associated with progressively larger benefits in terms of insulin sensitivity, glycemic control, lipid profile improvement, blood pressure reduction, and cardiovascular and renal risk prevention, as well as improvements in other weight-related metabolic and non-metabolic disorders [57]. Higher baseline body weight and BMI, compared with lower values, are generally associated with faster and more pronounced weight loss with current therapeutic strategies [58]. Similarly, individuals with a greater metabolic burden - such as those with moderate-to-severe hepatic steatosis, insulin resistance, and low muscle-to-fat or muscle-to-visceral fat ratios - are more likely to experience substantial weight loss during pharmacological treatment, including agents such as semaglutide and metformin [59].

Because patients with high baseline BMI and a substantial burden of obesity-related complications require intensive treatment to maximize weight loss in the short- and intermediate-term, and given that these individuals often experience rapid and marked weight reduction with current pharmacological strategies, they are at increased risk of skeletal muscle wasting and, in some cases, overt sarcopenia or residual SO. Consequently, promoting preferential FM loss while minimizing reductions in SMM is emerging as a novel and clinically relevant therapeutic target. Several real-life studies have evaluated the effects of pharmacologically induced weight loss in individuals with T2D who underwent body composition assessment according to prespecified protocols. Across follow-up periods ranging from 3 to 12 months, these studies have shown that glucagon-like peptide-1 receptor agonists (GLP-1RAs) and sodium-glucose cotransporter-2 inhibitors (SGLT2is) induce body weight reduction in more than 50% of treated individuals, predominantly through selective loss of total and visceral FM, with mild or moderate impact on SMM and no detectable impairment in handgrip (forearm) strength [60–62]. Most, but not all, participants had excess weight - defined as BMI > 25 kg/m² or waist circumference > 90 cm for men and > 80 cm for women - and experienced mild-to-moderate weight loss during follow-up while receiving semaglutide (predominantly 0.5 mg/week) and/or SGLT2is. Despite certain limitations, including a relatively small sample size, short duration of follow-up, and use of bioimpedance-based body composition assessment, these studies consistently indicate that weight loss induced by GLP-1RAs and SGLT2is is well balanced, with a more pronounced reduction in total and visceral FM compared with FFM and SMM. The findings of a meta-analysis of 19 clinical trials appear to support this observation, demonstrating that GLP-1RA therapy in individuals with T2D leads to substantial FM loss while only minimally affecting FFM [63]. Similar findings have been observed for SGLT2is [64]. However, data from specific trials involving patients with significant baseline obesity (e.g., BMI > 35 kg/m²) treated with high-dose GLP-1RAs (e.g., semaglutide 2.4 mg/week) or tirzepatide (up to 15 mg/week) have shown promising weight loss - often exceeding 10% of baseline body weight - primarily due to FM reduction. These studies also reported a notable decrease in lean mass (LM), accounting for up to 25% of the total weight lost [65]. LM loss appears to be even more pronounced with other agents, including semaglutide at standard doses (approximately 40%) [66]. This pattern is also observed in post-bariatric surgery patients, where the addition of incretin-based therapy compared with no pharmacological treatment further enhances weight loss, resulting from combined reductions in both FM (− 4.8 kg) and LM (− 3 kg) [67].

Most importantly, muscle mass, muscle quality, and muscle function are distinct yet interconnected dimensions of skeletal muscle physiology, essential for evaluating musculoskeletal health [68]. Muscle mass refers to the total quantity/volume of skeletal muscle tissue, measured through techniques such as dual-energy X-ray absorptiometry, magnetic resonance imaging, or more commonly bioelectrical impedance analysis, representing the structural bulk of myofibers organized into fascicles and encased by connective tissues, independent of fat or bone. In contrast, muscle quality assesses the intrinsic efficiency of skeletal muscle tissue beyond its size/volume, quantified as force or power output per unit mass (e.g., strength-to-mass ratio). Information on muscle quality incorporates data related to specific information about type of fibers, fat infiltration as expression of myo-steatosis, extracellular matrix integrity, and neural activation, characteristics usually impaired by aging regardless of skeletal muscle mass changes and informative about functional impairment [69]. Muscle function, meanwhile, captures dynamic performance in practical tasks, including maximal strength, power (force × velocity), endurance, and coordination, evaluated via dynamometry, chair-stand tests, or gait analyses. Muscle function translates skeletal muscle mass and quality in neuromuscular coordination, contraction dynamics, and metabolic resilience, serving as a critical biomarker for overall functional capability of an individual in terms of mobility, disability and frailty risk [70]. Functional outcomes - such as strength and mobility - represent key endpoints of skeletal muscle performance, bridging muscle mass, quality, and function to real-life clinical health metrics like independence and risk of falls. Muscle strength is regularly quantified through maximal voluntary isometric or isokinetic torque. The most common measurement method of handgrip strength is the handgrip dynamometry that reflects skeletal muscle force, integrating cross-sectional area (mass) and fiber contractility (quality). Handgrip strength normally declines by 1–2% annually after 50 years [71]. Mobility - assessed through gait speed (normal value > 0.8 m/s) [72], timed up-and-go test (normal value < 12 s), or Short Physical Performance Battery (normal value ≥ 8/12 points) - integrates lower-limb strength and power, balance, and coordination data [73]. Functional outcomes perform better than isolated quantity and quality measures as they well represent overall skeletal muscle performance (e.g., force production and locomotion efficiency) and are also associated with adverse outcomes. Clinically, interventions targeting strength (resistance training) and mobility (multimodal exercise) yield superior gains in quality-adjusted life years versus mass-focused approaches alone [74]. Loss of skeletal muscle mass, quality, and physical performance in T2D patients, older and frail individuals creates a vicious cycle, impairing glucose control due to impaired glucose uptake and utilization, worsened insulin sensitivity, and diminished glycogen storage, ultimately driving HbA1c elevation and high glucose variability [75]. This deterioration fosters chronic hyperglycemia, accelerating microvascular and macrovascular complications through inflammation, advanced glycation end-products, and oxidative stress [76]. Frailty amplifies these effects bidirectionally: poor metabolic control fosters muscle catabolism and sarcopenia, meanwhile skeletal muscle deterioration leads to frailty-driven mobility loss, perpetuating adverse outcomes [77]. Functional deficits (e.g., grip strength decline, timed up-and-go test > 12 s) signal independence loss, especially in older adults where age-related skeletal muscle impairment results from deterioration of neural drive and contractility. First-line interventions are necessary to attenuate or prevent sarcopenic risk with resistance exercise to optimize skeletal muscle trophism and stimulate hypertrophy with specific training sessions achieving 70–85% 1RM to be performed 2 to 3 times a week [78]. According to guidelines, leucine-rich protein (1.2–2 g/kg/day) diet and multimodal lifestyle approach to reinforce aerobic muscle metabolism and balance are recommended to yield 15–40% gains in short physical performance battery [79].

Overall, caution is warranted in interpreting the data presented. First, loss of SMM does not necessarily indicate impaired muscle strength, and clinically meaningful deficits in strength or function may not occur even in patients experiencing substantial skeletal muscle loss. Weight reduction can also be associated with apparent skeletal muscle shrinkage due to a marked decrease in fat infiltration within steatotic muscles, as described with tirzepatide [80]. This clinical phenomenon may be so pronounced as to result in significant anthropometric changes that are readily apparent on physical examination, while not constituting a pathological finding. Most trials did not assess muscle strength or functional performance alongside SMM and overall body composition changes over time. When muscle strength or functional tests were performed, the results generally did not show significant deterioration in skeletal muscle performance. Additionally, there is considerable variability in the methods used in clinical trials and real-world settings to assess body composition, which introduces uncertainty in interpreting the clinical significance of changes in individual body compartments [81] and in comparing the effects of different weight loss interventions on skeletal muscle outcomes [82]. Terminology further complicates interpretation. LM and FFM are often used interchangeably, yet they differ subtly. FFM represents total body mass minus fat, encompassing muscle, bone, organs, skin, and water, whereas LM represents total body mass minus fat but includes a small yet non-negligible amount of essential fat contained in cell membranes. Finally, a key distinction exists between lean or FFM and SMM, with the latter representing only the muscular component of FFM [83]. Nevertheless, the potential loss of SMM and strength has raised clinically relevant concerns in routine practice. Further investigation is ongoing, and several therapeutic strategies - including protein supplementation and regular physical exercise - have been proposed to mitigate FFM loss during anti-obesity treatment [84]. ActRII antibodies represent a recently developed class of drugs targeting the ActRII signaling pathway. Inhibition of receptor ligands has been shown to induce skeletal muscle hypertrophy, in addition to promoting bone formation and enhancing hematopoiesis [85–87]. Accordingly, ActRII blockade appears promising for conditions characterized by pronounced skeletal muscle wasting, including sarcopenia and muscle deterioration associated with surgery- or drug-induced substantial weight loss.

## Effects of weight loss-inducing drugs on muscle strength, function, and physical performance

GLP-1RAs and tirzepatide, more than SGLT2i, induce significant weight loss frequently exceeding 10% of baseline body weight in individuals with excess adiposity and T2D [88]. Weight-loss effects are primarily through appetite suppression, caloric deficit, or natriuresis, but their isolated impacts on muscle strength, physical performance, risk of falls, and disability are underexplored, revealing a potential conflict between cardiometabolic benefits and sarcopenia exacerbation risks [89, 90]. With regard to weight loss and body composition changes, GLP-1RAs and tirzepatide are expected to reduce both FM and FFM due to improvement of insulin sensitivity. Besides the overall effect on weight loss and body composition, semaglutide utilization is associated with amelioration of physical performance (e.g., significant increase in walk distance on 6-minute walk distance test), reflecting relevant functional gains [91]. SGLT2i are known to reduce body weight in a magnitude not exceeding that observed with incretin-based treatments, but are associated with significantly lower the risk of falls (23% to 44%) as an adjunctive effect to cardiovascular protection [92].

Despite positive results, several unknowns or emerging risks could be underestimated and should be mentioned. GLP-1RAs have posed sarcopenia concerns, as some observations reported relevant FFM deterioration or slight grip strength reductions and consequent intolerance to physical exercise while losing weight [93]. The effect can be the indirect consequence of adverse events (nausea, vomiting, significant appetite restraints) that can be particularly evident - thus, dramatically exacerbating weight loss - in older patients and those with background SO before GLP-1RA therapy initiation. Despite significantly lower weight loss with SGLT2is - compared to GLP-1RAs and tirzepatide - similar concerns have also been raised among gliflozin-users with most trials underpowered for skeletal muscle and functional endpoints [94]. While targeted studies are still needed to fully elucidate the impact of current anti-obesity treatments on skeletal muscle quantity, quality, and function, it is evident that future therapeutic strategies must prioritize the utilization of not only inducing and sustaining weight loss medications but also strategies to prevent or counteract skeletal muscle wasting and muscle strength deterioration or, ideally, promote muscle hypertrophy and functional gains.

## Pharmacological treatment to stimulate skeletal muscle trophism: focus on target therapies

Skeletal muscle is a highly dynamic system regulated by intricate molecular mechanisms. In addition to direct stimulatory pathways that promote myofibrillar protein synthesis and muscle hypertrophy, muscle growth is particularly evident when inhibitory mechanisms are suppressed. The most well-characterized inhibitors of muscle growth are myostatin and activins. ActRII mediates the catabolic effects of activin A, myostatin, and other peptides belonging to the bone morphogenetic protein/transforming growth factor-β family on skeletal myocytes [95]. Specifically, binding of myostatin or activin A to ActRII triggers phosphorylation of small mothers against decapentaplegic homolog (Smad) 2/3 proteins, which subsequently form a complex with Smad4 and translocate into the nucleus. Within the nucleus, this complex activates genes involved in muscle catabolism via the canonical Smad-dependent pathway [96]. Myostatin-mediated Smad activation also inhibits muscle protein synthesis by suppressing the AKT-mTOR signaling pathway, the key anabolic pathway that negatively regulates forkhead box O-dependent transcription of genes controlling the ubiquitin-proteasome system (Atrogin-1 and MuRF-1) [97, 98] and autophagy [99].

The most extensively studied activin type II receptors are ActRIIA and ActRIIB, initially recognized for their role in mediating activin signaling at the pituitary, organizing the positive feedback by which the gonads stimulate pituitary gonadotropin secretion [100]. However, this effect is clinically inconsistent and does not result in gonadal dysfunction or broader endocrine system imbalance [101]. When activated by myostatin or activin [102], ActRIIB initiates signaling through a Smad2/3-dependent pathway, which in turn promotes protein catabolism in skeletal muscle [103]. Myostatin also inhibits the AKT/mTOR/p70S6 signaling pathway, thereby suppressing protein synthesis and myoblast differentiation - both critical processes for muscle cell differentiation, myotube hypertrophy, and overall muscle growth [104]. Consequently, myostatin and related peptides act as physiological “brakes” on skeletal muscle hypertrophy, antagonizing the effects of muscle-promoting myokines, such as IL-6 [105].

**Fig. 2:**
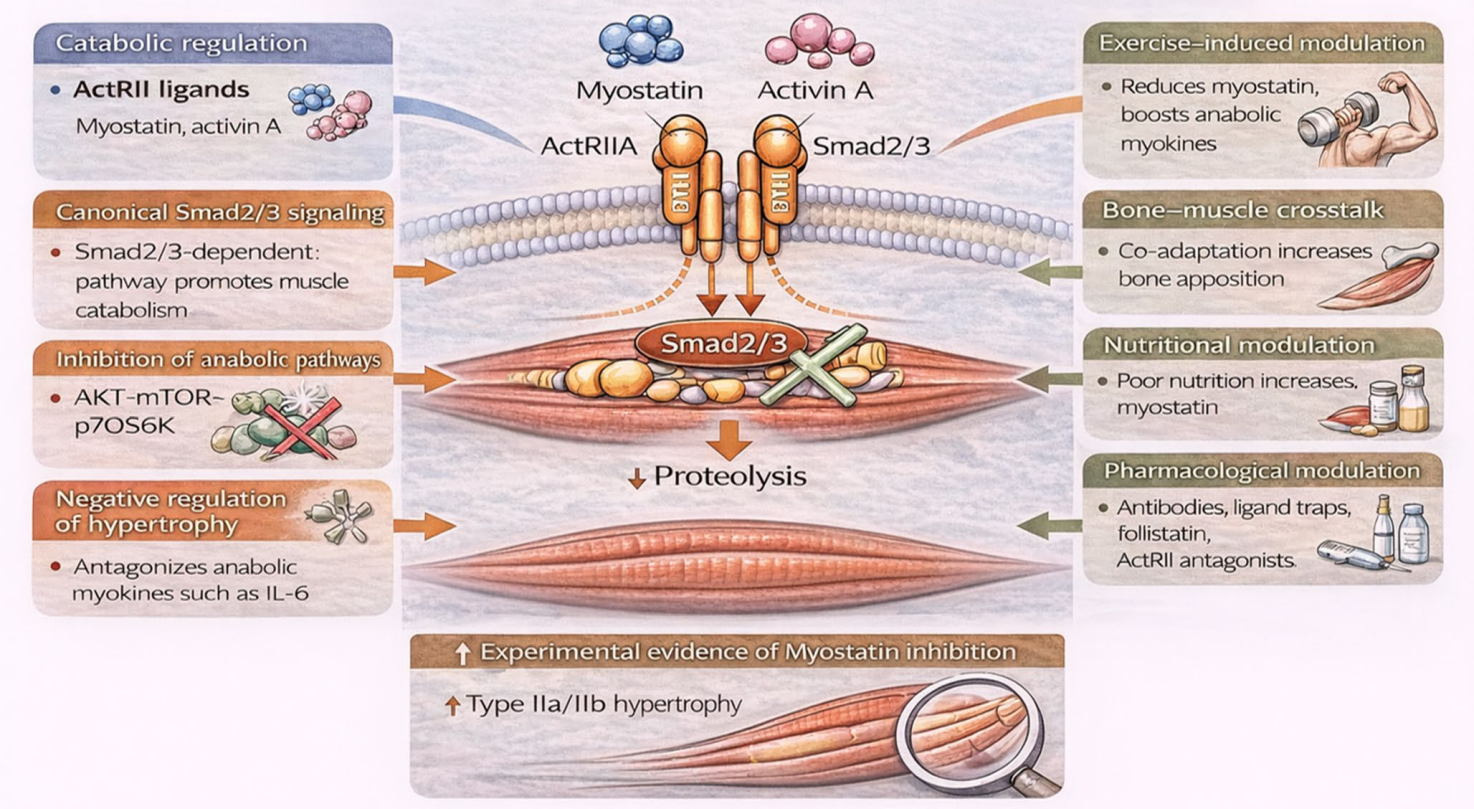
Illustration summarizing catabolic pathways, physiological modulation, and therapeutic implications for muscle mass regulation mediated by the ActRII/Myostatin signaling in skeletal myocytes [100–111]. Abbreviations: Activin Receptor type II, ActRII; Phosphorylated Protein Kinase B, AKT-P; mammalian Target of Rapamycin, mTOR; Ribosomal protein S6 kinase; p70S6K; Interleukin-6, IL-6; Small Mothers Against Decapentaplegic homolog, Smad

Physical exercise, particularly resistance training, is the most potent hypertrophic stimulus for skeletal muscle, promoting improvements in both strength and physical performance [106]. Acute bouts of resistance exercise have variable effects on circulating myostatin levels but consistently increase serum concentrations of follistatin - a direct myostatin inhibitor - and other muscle-promoting myokines for up to six hours or more post-exercise [107]. These molecular changes underpin skeletal muscle repair and regeneration following hypertrophic training and contribute to overall muscle adaptation to chronic exercise. Mechanistic studies further demonstrate that these processes are accompanied by bone tissue apposition at both trabecular and cortical sites in response to long-term physical training [108]. More recently, experimental evidence has shown that endurance exercise suppresses the expression of Ythdf1, a key regulator of myostatin mRNA. Loss of Ythdf1 leads to hyperactivation of skeletal muscle satellite cells, which play a critical role in muscle repair, hypertrophy, and regeneration following injury [109]. Conversely, as confirmed by both animal and human studies, food deprivation or insufficient intake of calories, protein, and carbohydrates significantly impairs muscle trophism and attenuates hypertrophic responses to exercise, likely mediated in part by increases in circulating myostatin levels (Fig. 2) [110, 111].

The central role of myostatin in suppressing skeletal muscle growth has been established by experimental studies since the 1990s. Silencing or antagonism of the myostatin gene results in pronounced, uncontrolled skeletal muscle hypertrophy - particularly affecting type IIA (intermediate glycolytic/oxidative) and type IIB (fast glycolytic/white anaerobic) fibers [112, 113] - through enhanced myofibrillar protein synthesis, myoblast differentiation [114], and myotube growth [115]. ActRII antagonism induces greater skeletal muscle hypertrophy than myostatin inhibition alone, as it interferes with multiple anti-hypertrophic signaling molecules, including myostatin, activin, and growth differentiation factor 11 [116]. Among selective ActRII-targeted approaches, simultaneous blockade of both ActRIIA and ActRIIB receptors is essential to maximize skeletal muscle hypertrophy [117]. However, while preclinical rodent models of ActRII inhibition robustly demonstrate muscle hypertrophy via myostatin/activin blockade [118, 119], human physiology exhibits equivocal responses due to differential ligand-receptor affinities (e.g., activin A predominance in primates over growth differentiating factor 8) [120] and compensatory pathways such as IGF-1 resistance in obesity, limiting translatability of results. Current mechanistic evidence relies heavily on correlative biomarkers with no longitudinal muscle biopsy data, while clinical trials reported variable effect on FFM and faced with limitations from short durations and heterogeneous obesity phenotypes [121]. Moreover, translational obstacles include ActRII traps’ off-target effects - such as cardiac hypertrophy, glucose dysregulation [122], and possible activin A-driven reproductive toxicities [123] - and skeletal muscle side effects (spasms), stalling earlier study programs such as in T2D [124]. A balanced approach is needed for addressing these systemic risks alongside combined regimens to optimize skeletal muscle preservation without compromising cardiometabolic safety.

Bimagrumab/BYM338 is a fully human monoclonal antibody which binds competitively to the ligand-binding domains of both ActRIIA and ActRIIB. By preventing ligand-induced activation of these receptors, bimagrumab promotes skeletal muscle hypertrophy by sparing myosin heavy chain from proteolytic degradation through inhibition of Smad2/3 phosphorylation [125], via a mechanism that is independent of the AKT/mTOR signaling pathway [126]. A randomized controlled trial published in 2014 demonstrated that bimagrumab increased both SMM and strength after 16 weeks of treatment in patients with sporadic inclusion body myositis, a myopathy characterized by overactivation of the Smad2/3 signaling pathway [127]. In a pilot study, Rooks and colleagues reported a marked increase in skeletal muscle volume together with substantial reductions in intramuscular and subcutaneous adipose tissue following administration of bimagrumab (30 mg/kg) in patients with casting-induced muscle atrophy [128]. Furthermore, a single intravenous dose of bimagrumab significantly increased LM (+ 2.7%) and reduced FM (−7.9%) in overweight, insulin-resistant individuals after 10 weeks of follow-up [129]. In a proof-of-concept study involving 40 community-dwelling individuals with sarcopenia, bimagrumab (30 mg/kg) induced significant improvements in both SMM and strength, assessed by bioimpedance analysis, gait speed, and the 6-minute walk test, respectively, with effects persisting for up to 16 weeks after treatment [130]. A subsequent dose-finding study reported comparable increases in LM and favorable changes in body composition in adults with obesity treated with either 30 or 3 mg/kg of bimagrumab after 4 weeks; however, these effects were sustained beyond 16 weeks only with the higher dose, indicating a dose-dependent durability of response [131]. More recently, a study comparing different routes of administration and dosing regimens evaluated the pharmacokinetics and pharmacodynamics of bimagrumab, including intravenous administration of 700 or 210 mg every 4 weeks; subcutaneous administration of 1500 or 525 mg every 12 weeks; and weekly subcutaneous doses of 300, 150, or 52.5 mg. After 20 weeks of treatment, improvements in LM and SMM were observed across all groups except those receiving the lowest dose (52.5 mg). Although subcutaneous administration resulted in approximately 40% bioavailability compared with intravenous delivery, pharmacodynamic outcomes did not differ significantly between weekly subcutaneous and monthly intravenous regimens, with comparable gains in LM (~ 2 kg) and reductions in FM (~ 2.5 kg) [132]. Evidence from the literature has yielded mixed results regarding the overall effects of bimagrumab on skeletal muscle strength and function. In a study by Heymsfield and colleagues, bimagrumab treatment, compared with placebo, resulted in a 20% reduction in FM, an 8.5-cm decrease in waist circumference, and a 5.7% reduction in body weight, alongside a decrease in liver fat fraction. These changes were accompanied by a 4.4% increase in LM and a modest improvement in bilateral handgrip strength over 48 weeks of treatment [133]. In contrast, other studies have failed to demonstrate meaningful improvements in muscle strength or functional performance with bimagrumab, despite substantial gains in lean and SMM compared with placebo, over treatment periods extending up to two years across different clinical conditions (Table 1) [134–137]. Despite the need for caution in interpreting available data [138], a systematic review and meta-analysis demonstrated that bimagrumab improves body composition but does not significantly enhance muscle strength or physical performance in patients with sarcopenia [139]. Furthermore, a systematic review evaluating the efficacy and effectiveness of a broad range of pharmacological interventions - including bimagrumab - reported no overall improvements in muscle strength, functional outcomes, or physical performance in individuals with inclusion body myositis [140]. Although substantial improvements in body composition have been observed up to three months following a single administration of bimagrumab, there is currently no convincing evidence supporting a beneficial effect of ActRII inhibition on muscle strength or physical performance. Well-designed, condition-specific studies are therefore required to better elucidate these effects, particularly in individuals with obesity and SO treated with bimagrumab alone or in combination with other anti-obesity pharmacotherapies and resistance exercise.

**Table 1:** The table summarizes clinical studies examining the effects of several doses and administration routes of bimagrumab on body composition and functional outcomes across various populations, including those with muscle atrophy and metabolic disorders

Reference	Study type	Population	Dose - regimen	Duration (weeks)	Variables	Body Composition Changes	Clinical meaning
Amato et al. [127]	RCT	Sporadic inclusion body myositis	One-shot (iv) 30 mg/kg vs. placebo	16	SMV, strength	↑ SM volume (+ 7%) on MRI ↑ 6MWD (+ 14.6%)	Muscle volume and function amelioration
Rooks et al. [128]	Double-blind, placebo-controlled trial	Casting-induced muscle atrophy	One-shot (iv) 30 mg/kg vs. placebo	12	SMV, IMAT	↑ SM volume (after 2 but not 12 weeks); ↓ Intramuscular and subcutaneous fat (−6.6% at 12 weeks)	Muscle volume improvement after 2-weeks of treatment
Garito et al. [129]	One-harm prospective study	Overweight - obesity, insulin-resistant individuals	One-shot (iv) 30 mg/kg	10	LM, FM	↑ LM + 2.7%; ↓ FM −7.9% BW unchanged	Body composition improvement
Rooks et al. [130]	Phase II, RCT, proof-of-concept study	Community-dwelling sarcopenia	One-shot (iv) 30 mg/kg vs. placebo	16	SMV, gait speed, 6MWT	↑ SMV (+ 7.7%) ↑ gait speed ↑ 6MWT	Muscle volume and function amelioration
Rooks et al. [131]	Dose-finding study	Older adults with obesity	30 mg/kg vs. 3 mg/kg	20	SMV, body composition, strength	↑ LM (both doses) but sustained at 30 mg/kg only No strength changes	Body composition improvement
Petricuol et al. [132]	Double-blind, placebo-controlled, parallel-arm, multiple-dose RCT	Older adults	IV: Cohorts 1–2, 700 − 210 mg q4w; SC: Cohorts 3–4, 1500 − 525 mg q4w; SC: Cohort 5, 300; Cohort 6, 150; Cohort 7, 52.5 mg weekly	20	FFM, FM, bioavailability	↑ LM/SMM (4–6%, except for the lowest dose); ↓ FM (~ 2.5 kg); SC administration: ~40% bioavailability	SC weekly administration provides comparable effective and safety effect with once-monthly SC and IV administration
Heymsfield et al. [133]	Phase II, RCT	T2D, overweight or obesity	10 mg/kg (up to 1200 mg) vs. placebo (5% dextrose solution) IV every 4 weeks up to 48 weeks	48	FM, FFM, waist, BW, liver fat, LM, HbA1c	↓ FM (−20%); ↓ waist (−8.5 cm); ↓ BW (−5.7%); ↓ Liver fat; ↑ LM (+ 4.4%) glucose control amelioration	Moderate weight loss, with relevant amelioration of body composition and glucose control. No functional effects

Results indicate significant improvements in skeletal muscle volume and reductions in fat mass, highlighting the intervention's potential to enhance quality of life and metabolic health. Less strengthened are results derivate from functional data, thus requiring further exploration of this endpoint

Randomized Controlled Trial, *RCT* Skeletal Muscle Volume, *SMV* Intramuscular Adipose Tissue, *IMAT* Lean Mass, *LM* Fat Mass, *FM* Body Weight, *BW* Six-Minute Walk Distance test, *6MWD* Fat-Free Mass, *FFM* Glycated Hemoglobin, *HbA1c* Type 2 Diabetes, *T2D* Subcutaneous administration, *SC* Intravenous administration, *IV* Every 4 Weeks, q4w

If future clinical trials will confirm the efficacy and safety of ActRII-targeted adjunct therapies, such as bimagrumab, these agents would be particularly relevant in high-risk populations vulnerable to skeletal muscle loss during weight reduction as stand-alone or add-on to current and future anti-obesity medications. Among such candidates, older adults with obesity and T2D with established diagnosis of SO should be prioritized to ActRII traps utilization, since this phenotype heightens cardiometabolic risks and frailty. Similarly, patients undergoing substantial weight loss via bariatric surgery or potent anti-obesity pharmacotherapies - who present with low SMM - stand to benefit from skeletal muscle preserving-intervention to mitigate functional decline and metabolic dysregulation. Also, individuals at high risk of falls or with pre-existing functional impairments can be another critical group, in which SMM depletion is highly expected to exacerbate postural control deficiency while standing or moving, and injury susceptibility.

## Discussion

For decades, the lack of long-term, effective, and safe pharmacological options to induce sustained weight loss and prevent weight regain in individuals with obesity has created a substantial therapeutic gap. This gap has resulted in a polarized management approach, characterized by reliance on basic nutritional counseling and general lifestyle interventions for most patients, with a direct transition to metabolic surgery reserved for severe or complicated cases. According to available estimates, only approximately 1% of eligible individuals have undergone surgical treatment for obesity, due to a combination of factors including patient and physician attitudes, economic barriers, perceived surgical risks and complications, and the complexity of long-term follow-up [141]. Consequently, for many years individuals living with obesity have received suboptimal, poorly structured, or inadequate weight management, predisposing them to failure in achieving durable weight loss, weight regain after initial success, and progressive development of chronic obesity-related complications [142]. Incretin-based therapies have substantially reshaped the treatment paradigm for obesity, as demonstrated by the long-term administration of semaglutide in the SELECT trial [143]. Tirzepatide has shown impressive outcomes in terms of weight reduction and sustained weight management in individuals with moderate-to-severe obesity [144], exceeding the effects observed with GLP-1RAs alone [145, 146]. In addition to promoting weight loss, incretin-based therapies have been shown to reduce the burden of obesity-related complications from metabolic [147], cardiovascular [148], and renal perspectives [149]. Despite the considerable costs associated with these medications - which represent one of the limitations to their long-term use in many countries - pharmacological management of obesity now constitutes an effective and safe strategy to bridge the longstanding gap between lifestyle interventions and metabolic surgery [150]. Current recommendations advocate for individualized therapeutic targets in individuals undergoing anti-obesity pharmacological treatment. Effective and safe weight loss should preferentially - ideally exclusively - consist of FM reduction, with particular emphasis on visceral adipose tissue [151], whose excess represents a major determinant of adverse health outcomes [152]. Anti-obesity interventions, including dietary strategies and pharmacological therapies, effectively target the adipose tissue, especially visceral depots; however, they are also associated with concomitant reductions in FFM and SMM, which - according to available estimates - may account for approximately 20–50% of total weight loss [153]. Although the clinical significance and interpretation of these body composition changes remain to be well established and warrant further investigation, there is a clear need to support skeletal muscle health in individuals living with obesity, particularly those with marked baseline body weight excess. While protein supplementation and regular physical exercise are expected to preserve or enhance SMM during weight loss, no dedicated studies or structured intervention protocols combining specific lifestyle strategies with pharmacological or surgical treatments have been published so far. Consequently, skeletal muscle wasting - and its potential impact on muscle strength during weight reduction - remains an important clinical concern, especially in individuals experiencing substantial weight loss.

The pharmacological pipeline for obesity is highly active, with numerous agents under investigation targeting multiple mechanisms, including enhanced appetite suppression, increased satiety, skeletal muscle preservation, and adipose tissue browning [154–160]. Among these, the human monoclonal antibody bimagrumab, an activin type II receptor antagonist, has shown promising results across a wide range of skeletal muscle disorders. In patients with obesity and SO, bimagrumab has been demonstrated to preserve SMM and improve body composition by increasing the skeletal muscle-to-FM ratio during weight loss interventions [161]. The existence of AKT-independent pathways, which promote myofibrillar protein synthesis while inhibiting proteolysis and autophagy in skeletal muscle cells, provides a strong mechanistic rationale for the use of ActRII inhibitors in individuals with obesity undergoing hypocaloric diets, either alone or in combination with incretin-based anti-obesity therapies such as semaglutide and tirzepatide. However, uncertainties remain regarding the effects of bimagrumab on muscle strength and functional performance, whether administered alone or alongside other anti-obesity medications. Well-designed randomized controlled trials are required to address these specific questions, and additional studies are necessary to evaluate potential cardio-renal-metabolic benefits.

Older adults with T2D and excess adiposity represent a particularly vulnerable group in whom muscle loss develops earlier, progresses faster and carries significant functional consequences than normal. These observations reinforce the need for age- and sex-driven strategies that go beyond weight reduction alone and specifically target preservation of SMM and function in patients with T2D and obesity. Finally, age and sex influence the trajectory of SMM decline independently of comorbidities. Individuals with prediabetes and T2D exhibit a more pronounced reduction in appendicular FFM, SMM, and muscle strength compared with metabolically healthy counterparts. This phenomenon becomes particularly evident at older ages, in parallel with the development of anabolic resistance to insulin, reduction in physical activity levels, and progressive neuromuscular dysfunction [162]. While chronological age remains a major determinant of sarcopenia, both diabetes duration and age at disease onset emerge as independent modifiers of risk, indicating that early metabolic derangements exert persistent detrimental effects on skeletal muscle health. In recent decades, the earlier onset of T2D [163] has been associated with a higher prevalence of reduced muscle mass and strength compared with age-matched individuals with later-onset disease. Prolonged exposure to hyperglycemia, insulin resistance, and chronic low-grade inflammation from a younger age is likely to accelerate muscle protein breakdown and impair anabolic signaling well before the onset of physiological age-related muscle decline. In this context, early-onset T2D can shift sarcopenia from a predominantly geriatric condition to a midlife complication, thereby increasing the cumulative lifetime risk of functional impairment [164]. Sex-specific differences further modulate the complex interplay between diabetes, obesity, and muscle loss through hormonal, metabolic, and body composition-related mechanisms. Although both men and women with T2D show a higher prevalence of sarcopenia and a steeper decline in total, truncal, and appendicular FFM compared to healthy controls, lower baseline muscle reserves and the loss of estrogen-mediated protection after menopause exacerbate sarcopenia-related outcomes in women [165]. These observations underscore the need for dedicated studies and age- and sex-specific therapeutic strategies extending beyond weight reduction alone and prioritizing the preservation of SMM and strength and physical performance in individuals with T2D and obesity.

## Conclusion

While incretin-based therapies have revolutionized obesity management by delivering sustained weight loss and mitigating cardiometabolic complications in patients with obesity - and such specific phenotypes like SO and aged individuals - and T2D, current concerns about their potential with substantial FFM and SMM highlights an unmet need to preserve skeletal muscle health (quality, quality, and function), particularly in vulnerable populations. ActRII traps, such as bimagrumab, offer a mechanistically fitting adjunctive strategy to enhance FFM and SMM retention, improve body composition, and potentially preserve functional outcomes during potent anti-obesity interventions, both pharmacological and surgical. Future RCTs are essential to confirm these benefits, optimize combination regimens (as adjunct to physical exercise and polypharmacotherapy), and establish cardio-renal-metabolic synergies, thereby enabling a paradigm shift toward fat-preferential and muscle-sparing weight loss.

## Data Availability

No datasets were generated or analysed during the current study.

## Abbreviations

ActRII: Activin Receptor type II
BMI: Body Mass Index
FM: Fat Mass
FFM: Fat-Free Mass
GLP-1RAs: Glucagon-Like Peptide-1 Receptor Agonists
GH: Growth Hormone
IGF-1: Insulin-like Growth Factor 1
IL: InterLeukin
LM: Lean Mass
mTOR: Mammalian Target of Rapamycin
AKT: Protein kinase B
SO: Sarcopenic Obesity
SMM: Skeletal Muscle Mass
SGLT2is: Sodium-Glucose (co)Transporter-2 inhibitors
Smad: Small Mothers Against Decapentaplegic homolog
TNF-α: Tumor Necrosis Factor-α
T2D: Type 2 Diabetes
